# The relationship between enterprise human capital structure and international M&A performance based on cultural differences in China

**DOI:** 10.1371/journal.pone.0289270

**Published:** 2025-03-10

**Authors:** Ye Liu, Fang-yuan Zhao, Si-qi Jia, Li-rong Qiu

**Affiliations:** School of Business Administration, Northeastern University, Shenyang, Liaoning, China; Yunnan Technology and Business University, CHINA

## Abstract

With the rapid development of China’s economy, increasing Chinese companies have acquired international technology resources, improved production efficiency, opened up international markets through international mergers and acquisitions, and realized the expansion of enterprises in the global capitalist market. Especially in recent years, the rapid development of international mergers and acquisitions of Chinese enterprises, as well as the increasingly frequent activity in the international market, makes the performance of international mergers and acquisitions become the focus of attention of enterprises, and also increasingly become a focus of scholars. Although the human capital structure of enterprises as an internal factor of enterprises has been studied by most scholars. However, in the existing literature, more studies focus on the impact of corporate human capital structure on corporate performance, while the impact of human capital on international M&A performance is not sufficient. The performance of international mergers and acquisitions is relatively a more complex factor, which is influenced by more factors and has higher research value. Based on the performance of international mergers and acquisitions, this paper explores how the human capital structure of enterprises can play a role in improving the competitiveness of enterprises under the background of economic globalization, so as to provide reference for enterprises to gain competitive advantage in the international market. This study selects Chinese listed companies with international mergers and acquisitions from 2008 to 2015 as research samples, and empirically analyzed the relationship between corporate human capital structure and international M&A performance. The four conclusions obtained are as follows: (1) Employee compensation, employee education level and employee technical personnel ratio are significantly positively correlated with the company’s international M&A performance; (2) Enterprise training in both short-term and long-term. Neither of them has a important impact on the company’s international M&A performance; (3) Employee turnover has a critical negative impact on the company’s international M&A performance; (4) After introducing cultural differences as a regulatory variable, it is found that cultural differences can weaken the impact of human capital factors on the performance of international M&A. Finally, this paper puts forward suggestions on how to better utilize the human capital advantages of the company in the global economic background with the intention of improving the performance of international M&A.

## 1. Introduction

### 1.1. Research background

Since China’s entry into WTO, it has become more and more closely connected with other countries in the world. Under the background of economic globalization, international mergers and acquisitions have gradually arisen, developed rapidly and experienced many upsurge. Chinese foreign investment M&A activities are becoming more and more active, and more and more Chinese enterprises begin to explore overseas markets, and international M&A has become one of the important ways of international investment development. A series of cases, such as Geely’s acquisition of Volvo and Lenovo’s acquisition of IBM, all show that Chinese enterprises have strong capital strength and have the ability to occupy a position in the world capital market. However, with the arrival of the financial crisis in 2008, international M&A enterprises have encountered unprecedented challenges, and the performance of international M&A has become a topic of concern and discussion. People find that not all enterprises can benefit from international M&A, especially in the downturn of the international economic environment, the survival of international M&A enterprises is facing unprecedented challenges. Therefore, how to make use of international resources to achieve their own rapid development under the background of global economy has become the main goal of research.

Since entering the era of knowledge economy, human capital has become a research hotspot, and its role has been paid more and more attention. In 1960, Schultz, winner of Nobel Economics, put forward the theory of human capital systematically for the first time, which proved that compared with other capitals, human capital will bring higher return on investment. Human capital, as one of the important components in an enterprise, refers to the sum of quality stocks embodied by each individual. Different enterprises have different knowledge and abilities, which determines the different market positions of enterprises. Especially in the context of globalization, higher quality human capital can promote the internationalization of enterprises, thus making enterprises gain certain advantages in the tide of international mergers and acquisitions.

In recent years, the role of culture in national development has become increasingly prominent, and culture plays an indispensable role in improving comprehensive national strength. The degree of cultural differences between the two sides of mergers and acquisitions will affect the performance of mergers and acquisitions to a certain extent. Studies have found that mergers and acquisitions can make the shareholders of the acquired enterprises gain profits to a certain extent, but for most mergers and acquisitions enterprises, mergers and acquisitions can not effectively enhance the potential value of enterprises, on the contrary, it will significantly reduce the performance level of enterprises. As to why M & A can not create value for M & A enterprises, the degree of cultural differences between M & A parties plays an important role. The existence of cultural differences between the two sides of mergers and acquisitions will lead to internal management disorder and personnel mobility after mergers and acquisitions, which makes enterprises unable to achieve profit goals in a short time.

Based on this, this paper puts forward the research problem of the relationship between enterprise human capital structure and international M&A performance based on cultural differences.

### 1.2. Research significance

Human capital is an important part of enterprise resources, which affects many aspects of enterprises, including the performance of mergers and acquisitions. Under the background of globalization, having deeper human capital is usually considered to enable enterprises to occupy a more favorable position in international competition. This paper studies the relationship between human capital and international M&A performance by taking Chinese listed companies with international M&A as research samples and introducing cultural differences as moderating variables, which has the following research significance:

(1) Through the research on the structure of human capital, the empirical analysis method is used to test the relationship between human capital and the performance of international mergers and acquisitions, and to understand and analyze the influence mechanism of human capital on the performance of international mergers and acquisitions, so as to provide ideas for the cultivation of human capital and the management of human resources in enterprises.
(2) Introducing cultural differences as a regulatory variable, and then under the regulatory mechanism of cultural differences, we can have a deeper understanding of the influence path of enterprise human capital on international M&A performance, and put forward feasible policy suggestions according to the conclusion of empirical analysis.

### 1.3. Research innovation

Previous scholars have studied the relationship between human capital and corporate performance, and there are few studies linking human capital, cultural differences, and international M&A performance. We divides the human capital structure of the enterprises into five dimensions: employee compensation, employee training investment, employee education level, employee turnover, and technical staff ratio. The cultural differences among countries are used as the moderator variable to study the relationship between corporate human capital and international M&A performance. The two innovations of our paper are as follows: Firstly, we not only intends to divide the human capital structure into five dimensions, including employee compensation, enterprise employee training investment, enterprise employee education level, enterprise employee fluidity, and technical staff ratio, but also build human capital and international M&A performance model, which separately study its impact on international M&A performance, and provide new ideas for studying the issue of Chinese M&A performance. Secondly, we intends to use adding the cultural differences as a moderator to the model of human capital structure and international M&A performance, and study the impact of Chinese corporate human capital structure on international M&A performance under the influence of cultural differences. The addition of cultural differences regulating variables fills the gaps in previous research.

### 1.4. Research framework

This study is divided into the following five parts:

The first part is the introduction. This part mainly includes the following contents: the research background, research significance, research innovation and the organizational structure of this paper.

The second part is literature review and research hypothesis. First of all, this paper collates and analyzes the literature review of the related issues studied in this paper, and puts forward the research hypothesis of this paper.

The third part is variable design and model construction. Firstly, the variables are designed, and the measurement methods and calculation formulas of each index are explained. Secondly, a theoretical regression model is constructed according to the proposed assumptions.

The fourth part is empirical analysis. Including data collection and empirical test, first of all, explain the source of sample selection and brief data processing. Secondly, using SPSS 21.0, Stata 15.0 and other measurement software, regression analysis and correlation test were carried out on the samples. Finally, the robustness test is carried out with measurement software to ensure the validity of data and the accuracy of results.

The fifth part is the conclusion and suggestion. This part mainly includes the research conclusions, policy recommendations, research limitations and the prospect of future research directions.

## 2. Literature review and theoretical assumptions

### 2.1. Employee compensation and international M&A performance

Since the human capital has been attracted attention, as one of the important measures of human capital, the relationship between employee compensation and performance has been studied by many scholars. We mainly explores the impact of employee’s average wage on international M&A performance. Most studies has confirmed that employee wage is positively related to corporate performance. High salary can not only motivate employees to improve labor productivity, but also effectively retain excellent talents,reduce the turnover rate, and then decrease the cost caused by employee turnover. Meanwhile, high salaries can introduce high-quality talents from outside the company, improve the quality of human capital, and enhance corporate performance. Perfect salary system is an important means for enterprises to compete for talents and create value [1].pointed out that in the short term the the increase of salary would help improve corporate performance [2].found that the positive effects of employee compensation on business performance were particularly evident by studying five hotels in Indonesia [3].took the manufacturing listed companies in Shanghai and Shenzhen stock markets in China from 2009 to 2013 as samples, and investigated the salary of ordinary employees from three aspects: per capita salary, salary gap and salary growth rate, and studied the relationship between it and enterprise performance. Through empirical research, it is concluded that the improvement of ordinary employees’ salary has a positive role in promoting enterprise performance, and compared with high-income employees, the salary incentive effect is more obvious for low-income employees [4].took 1554 A-share listed companies in Shanghai and Shenzhen from 2012 to 2016 as samples (including 837 private enterprises and 717 state-owned enterprises). The results show that there is a close relationship between salary structure and enterprise performance [5].studied the relationship between executive-executive and executive-employee salary gap and enterprise performance. Taking A-share listed companies in China from 2012 to 2014 as samples, through empirical analysis, it is concluded that reasonable employee salary gap can stimulate the work enthusiasm of executives and employees, thus further improving enterprise performance. On the contrary, unreasonable salary system will make employees feel dissatisfied and cannot play an incentive role, thus having a negative impact on enterprise performance [6].divides employee salary into fixed salary and variable salary. This paper selects the relevant data of A-share listed public enterprises from 2009 to 2016, and points out through empirical research that executive variable salary can improve enterprise performance, while employee fixed salary can improve enterprise performance.Through empirical researches, it was found that, in generally, employee compensation had a positive impact on corporate performance. but when employee compensation gap was large, the existence of salary rigidity had a negative impact on business performance. Based on the above analysis, this paper proposes:

Hypothesis 1: Corporate employee compensation is positively correlated with international M&A performance.

### 2.2. Staff Training and International M&A Performance

Staff training is an important means of accumulating human capital. The training of employees not only enhances their own physical ability, but also enables employees to fully understand the company and master the knowledge and skills required in their work to making the operation of the company more efficient.

[7] used a variety of measurement methods to study the impact of employee training on enterprise productivity. The results show that an increase of 1% in employee training will increase enterprise labor productivity by 0.6%. [8]studied the two-way relationship between employee training and enterprise performance from the perspective of enterprises. By analyzing the data of Chinese manufacturing enterprises from 2004 to 2007, it is concluded that there is a two-way relationship between employee training and enterprise performance. On the one hand, staff training makes the performance indicators of enterprises better than those without staff training, and the risk of business failure will decrease after staff training. On the other hand, the better the enterprise, the more inclined it is to provide training for its employees, and the investment in training will increase the number of employed people. [9] studied the impact of employees’ on-the-job training on enterprise performance. Empirical research shows that the impact of on-the-job training on enterprise performance is diversified. Compared with those enterprises that are difficult to invest in on-the-job training, those enterprises that are easy to invest in on-the-job training have a more significant effect on improving enterprise performance, and the output effect of on-the-job training is higher than the wage increase effect of employees.Foreign scholars, such as [7,10–12]and others, have also reached the same conclusions through empirical research, which is that there is a positive correlation between employee training and corporate performance. When employee training increases, corporate performance will also improve. Based on the above analysis, this paper proposes:

Hypothesis 2: Corporate employee training investment is positively correlated with international M&A performance.

### 2.3. Employee education level and international M&A performance

Education can reflect the quality and ability of a person, which is an important part of human capital. And receiving education is a significant way to form human capital. The overall education level of employees can reflect the overall quality level and human capital level of employees. It is generally believed that the higher the education level of the employees, the higher the quality of the human capital of the enterprise. The quality of the human capital and the quality of the employees of the enterprise play an important role in the development of the enterprise. As can be seen from most of the literature at home and abroad, most scholars believe that the education level of employees has a positive impact on corporate performance and international M&A performance.

[13] used the manufacturing enterprise as a research sample, and found through dynamic research that there was a positive correlation among the academic structure of employees and corporate performance. [14] took Chinese industrial enterprises from 2004 to 2005 as samples, used the education level of employees to measure the general human capital, and used the training investment of enterprises to employees as the exclusive human capital, and studied the impact of these two on enterprise performance. The results show that the employee education level has a significant positive impact on enterprise performance, the relationship between employee training investment and enterprise performance is “inverted U”, and there is a negative interactive effect between employee education level and employee training investment, that is, when the employee education level is low, employee training will have a more obvious impact on enterprise performance. [15]pointed out that the education level of employees is significantly positively correlated with enterprise performance when studying the impact of technological innovation on enterprise performance with the education level of employees as the regulating variable. In addition, many scholars take the education level of employees as a factor index of enterprise human capital structure to study its influence on enterprise performance, and find that there is a correlation between the education level of employees and enterprise performance, such as [16,17]and so on.Based on the above analysis, this paper proposes:

Hypothesis 3: The education level of employees is positively related to the performance of international M&A.

### 2.4. Employee mobility and international M&A performance

Compared with physical capital, human capital is proactive and its flow process is more complicated [18].wrote that “Depending on the research perspective, employee mobility can have different classifications:

(1) active mobility and passive mobility, based on the subjective will of the mobile personnel themselves. Active mobility includes retirement, health reasons, resignation, etc. Passive mobility includes layoffs, dismissals, expulsions, contract expirations, demotions, work-related injuries, deaths, etc.;
(2) Functional mobility and dysfunctional mobility are based on the performance of employee mobility, the willingness of the organization to continue to employ him, and the ease of replacing him;
(3) Explicit mobility and implicit mobility, implicitly flowing employees have a mobility intention, engaging in things that are not related to their own work, but still maintain the state of contractual relationship. The normal flow of employees helps to promote the survival of the fittest and optimize the allocation of human resources, which is conducive to the long-term development of the enterprise and beneficial to the enterprise. On the contrary, frequency and abnormal employee turnover will result in the loss of human capital investment and increase the replacement cost of enterprise talents. At the same time, the loss of excellent talents will affect the improvement of the quality of the entire enterprise staff, while the loss of important management and technical personnel means the loss of enterprise intangible assets, such as customer list and enterprise technology, which will reduce the competitiveness of enterprises and affect the performance of enterprises. In the existing literature, most scholars have studied the impact of employee flow on enterprise performance through turnover rate [19].studied the impact of employee turnover rate on business performance. Empirical analysis showed that employee turnover rate significantly affected the profitability of the company. The higher the employee turnover rate, the worse the profitability of the company [20,21]. and others also reached the same conclusion. Based on the above analysis, we proposes:

Hypothesis 4: Corporate employee mobility is negatively correlated with international M&A performance.

### 2.5. Technical staff ratio and international M&A performance

Technology (including R&D) personnel is the core of the enterprises and is the main force for providing technological innovation to enterprises. Technological innovation is an important innovation capability of enterprise human capital. If enterprises want to be in a dominant position in the market, they must develop technology and drive technological progress. Therefore, as a reflection of the company’s innovation ability, technical professional human capital is an important part of enterprise human capital [22].used the proportion of technicians as one of the indicators of corporate human capital, and studied the relationship among them and corporate performance. The study found that the proportion of technicians was significantly positively correlated with corporate performance [23].selected durable goods manufacturing enterprises as samples to study the relationship between employee skills and enterprise performance. The analysis results show that there is a relationship between them, and this relationship is systematic [24].selected the data of 506 high-tech enterprises in Italy, and studied the relationship between human capital and enterprise development. The article pointed out that the proportion of technical personnel in enterprises is helpful to promote the development of enterprises [25].measures human capital from two dimensions: educational background composition (bachelor degree or above, bachelor degree or below) and professional structure (sales staff, technical staff, production staff and administrative staff), and studies the relationship between human capital and M&A performance. By collecting the data of A-share listed companies that acquired from 2007 to 2009, it is concluded that there is a significant correlation between the proportion of technical staff and M&A performance, and the proportion of technical staff negatively affects M&A performance. The reason may be that in the process of M&A, the ability of technical staff is not well integrated, which makes the technical synergy effect after M&A not well exerted.Based on the above analysis, this paper proposes:

Hypothesis 5: The proportion of employees in the company’s employees is positively related to the performance of international M&A.

### 2.6. The adjustment of cultural differences

[26] pointed out that “cultural differences are the level of difference among countries in terms of cultural norms”. Cultural differences refer to differences in religious beliefs, values, customs, and languages among different regions, nations, and countries.

After the international mergers and acquisitions of enterprises, due to the cultural differences between the two companies in terms of values and customs, the enterprises need to integrate internally, and the integration may cause the flow of employees, such as the mobilization of positions, the recruitment of talents, and the departure of personnel [27]. survey concluded that 58% of senior executives of acquired companies left the company within five years; the results of [28]also showed that the turnover rate of employees in the first year of the M&A was 26%. In the third year, the cumulative total was 49%. In the five years of M&A, the company’s employee turnover rate exceeded 50%, the manager’s turnover rate was 61%, but the normal employee turnover rate was only 5%. According to P·Pritchett and D·Robinson’s survey found that senior managers and technicians had a turnover rate of 47% in the year after the M&A, and the turnover rate reached 72% in the three years after the M&A. In a study of the impact of national cultural differences and organizational cultural differences on the turnover rate of executives in M&A [29],surveyed 270 US companies that were acquired by domestic and foreign companies from 1986 to 1988. It was found that the turnover rate of American companies acquired by foreign companies was higher than that of US companies acquired by domestic companies. This is because the difference between multinational organizations was greater than the difference between domestic companies, and it was more likely to cause personnel fluctuations. These documents indicate that the existence of cultural differences between M&A both sides will result in the loss of personnel, especially the departure of the core management of the enterprise, which will affect the emotions and psychology of the rest of the employees, making them absent-minded, reducing work efficiency, and thus affecting corporate performance.

[30] pointed out that cultural differences would bring problems in human resource management to multinational enterprises, such as employee recruitment, employee compensation, uncoordinated relationship between managers and ordinary employees, communication barriers within enterprises, etc., all of which would increase the management difficulty and the cost of enterprises and affect the development of enterprises. The article also points out that “cultural differences have a greater impact on employee compensation. Employees in different countries and regions have different expectations for compensation. There may be differences in the basis for the issuance of employee compensation. International M&A companies need to fully consider the means of payment for employees in the subordinate enterprises.

In the existing literature, more studies focus on the impact of corporate human capital structure on corporate performance, while the research on the impact of human capital on international M&A performance is not sufficient. As for the cultural differences between the two sides of mergers and acquisitions as a moderating variable, introducing the model of human capital structure and international M&A performance to study the impact of human capital structure of Chinese enterprises on international M&A performance based on the regulatory mechanism of cultural differences, the introduction of cultural differences moderating variables is also a vacancy in previous studies.

Based on the above analysis, we propose the following assumptions

Hypothesis 6: Cultural differences can reduce the positive impact of employee compensation on international M&A performance.

Hypothesis 7: Cultural differences can reduce the positive impact of employee training investment on international M&A performance.

Hypothesis 8: Cultural differences can reduce the positive impact of employee education on international M&A performance.

Hypothesis 9: Cultural differences increase the negative impact of employee flow on international M&A performance.

Hypothesis 10: Cultural differences can reduce the positive impact of employee-to-skills ratios on international M&A performance.

## 3. Variable design and indicator system

### 3.1. Explained variable

The explained variableof our paper are the international M&A performance of enterprises. In the existing literature, the measurement methods of international M&A performance mainly adopt event research method and accounting research method. Among them, the measurement index of event research method was the cumulative excess return of the stock market, which was mainly used to study short-term M&A performance; Accounting research method measures the financial indicators of enterprises, which is more effective in measuring the long-term M&A performance. Because we studies the impact of human capital based on cultural differences on the performance of Chinese companies’ international M&A, it involves the interaction between enterprises and host country stakeholders. According to the research of [31,32], we selects ROA of total return on assets as a measure of international M&A performance.

### 3.2. Explanatory variable

The explanatory variable of our paper is human capital. Divided into the following dimensions:

(1) Employee wage (WAGE). Our paper is based on the research of Ren et al. The wage level of the employees of the company is measured by the per capita wage of the employees. This value is derived from the ratio of “total compensation” in the company’s annual report to the “total number of employees”.
(2) Staff Training (TRA). Our paper is based on the research of Li Fubing et al. The investment in employee training is measured by the expenditure of training expenses per employee. This value is derived from the ratio of “employee training funds” and “total number of employees” in the annual report.
(3) Employee Education Level (EDU). We use Fan Qingliang and other people’s views to measure the education level of employees through the average years of education. Among them, the employee education is 3 years for the junior college education, 4 years for the undergraduate education, 7 years for the postgraduate education, and 10 years for the doctoral education. This value is obtained by multiplying the number of persons in each educational level by the ratio of the sum of the corresponding education years and the “total number of employees in the enterprise”.
(4) Employee flow (EF). According to the research of Luo Jia et al., we measures the employee turnover of the enterprises through the employee turnover rate. The formula for calculating the value is: (the total number of employees in the year of the international M&A event - the total number of employees in the previous year of the M&A event)/ The total number of employees in the previous year in the event of a merger.
(5) Technical Staff Ratio (TSRA). According to the research of Yin Feiyang et al., the proportion of technicians in the enterprise is also one of the indicators of human capital. This value is obtained from the ratio of “number of technicians” and “total number of employees” in the annual report.

### 3.3. Moderator variables

We takes cultural differences as a moderating variable. Most of the existing literatures used the Hofstede index to measure cultural differences between countries. According to the research of [33], we firstly obtains the values of each dimension index of the sample host country and China from Hofstede index website,calculates the six average scores, and then calculates the absolute value of the difference between the two countries as a measure index of cultural difference. The larger the index value, the greater the differences in the human environment between the two places. The specific calculation formula is:


$$CD_{j}=\left|\sum_{\text{i}=\text{1}}^{\text{6}}I_{i\text{j}}/6-\sum_{\text{i}=\text{1}}^{\text{6}}I_{in}/6\right|i=1,2,3,4,5,6;j=1,2,$$ (1)

Where CD_j_ refers to the cultural differences between China and J, I_ij_ is the score of country j in the i_-th_ dimension of national culture, and I_in_ represents China’s score in the i_-th_ dimension of national culture.

### 3.4. Control variables

In addition to the variables mentioned above, there are still some variables that have an important impact on international M&A performance. We use these variables as control variables, including enterprise size, enterprise age, international M&A experience of enterprises, industry relevance of M&A, shareholding ratio of first shareholder, nature of equity, and pre-merger performance. Variable definitions and descriptions are shown in Table 1.

**Table 1:** Definition of Related Variables.

	Variable	Variable symbol	Measurement index (calculation formula)
Explained variables	M&A performance in the year	Y0	ROA in the year of M&A
	The first year performance after M&A	Y1	ROA in the first year after M&A
	The second year performance after M&A	Y2	ROA in the second year after M&A
	M&A performance changes in the year compared to the previous year	Y01	Y0-ROA in the year before M&A
	Performance change in the first year after M&A relative to the year before M&A	Y11	Y1- ROA in the year before M&A
	Performance change in the second year after M&A relative to the year before M&A	Y21	Y2- ROA in the year before M&A
Explanatory variables	Employee wages	WAGE	Average wages: total wages/ total number of employees
	Staff training expenses	TRA	Average expenses: employee training expenses/ total number of employees
	Employee education	EDU	Average education years: (junior college number * 3 + undergraduate number * 4 + postgraduate number * 7 + doctoral number * 10)/total number of employees
	Employee fluidity	EF	(total number of employees in the year of M&A - total number of employees in the year before M&A)/total number of employees in the year before M&A
	Technical staff ratio	TSRA	Number of technicians/total number of employees
Moderator variable	cultural difference	CULD	Cultural differences between the country in which the target company is located and China (Hoffsted Index): the absolute value of the average phase subtraction of the six indices of Hofstede between the two countries
controlled variables	The enterprise size	LNSI	Log(Number of employees in the year before M&A)
	Enterprise age	AGE	Time from establishment to M&A
	M&A experience	EXP	Number of M&A before finishing M&A, Three times or more by 1, instead of 0
	Industry relevance	INDUS	Whether the merger enterprise is related to the target enterprise industry, if the correlation is denoted as 1, otherwise it is 0
	Proportion of holding stock	FH	The shareholding ratio of the largest shareholder in the year before M&A
	Corporate equity type	CSTYP	Whether the enterprise is a state-owned enterprise, if it is, the value is 1, otherwise it is 0.
	Pre-M&A performance	PIMAP	ROA in the year before M&A

### 3.5. Model construction

Based on the research of related literature, the following linear models are established:

Model 1: Employee wage and international M&A performance


$$Y_{t}=\beta_{0,t}+\beta_{1,t}WAGE+\sum \beta_{i,t}Control_{i}+\varepsilon_{t}$$ (2)

Among them, Yt is Y0, Y1, Y2, Y01, Y11, Y21.

Model 2: Staff Training and International M&A Performance


$$Y_{t}=\beta_{0,t}+\beta_{1,t}TRA+\sum \beta_{i,t}Control_{i}+\varepsilon_{t}$$ (3)

Model 3: Employee Education Level and International M&A Performance


$$Y_{t}=\beta_{0,t}+\beta_{1,t}EDU+\sum \beta_{i,t}Control_{i}+\varepsilon_{t}$$ (4)

Model 4:Employee fluidity and international M&A performance


$$Y_{t}=\beta_{0,t}+\beta_{1,t}EF+\sum \beta_{i,t}Control_{i}+\varepsilon_{t}$$ (5)

Model 5: Technical Staff Ratio and International M&A Performance


$$Y_{t}=\beta_{0,t}+\beta_{1,t}TSRA+\sum \beta_{i,t}Control_{i}+\varepsilon_{t}$$ (6)

Model 6: Corporate Human Capital and International M&A Performance


$$Y_{t}=\beta_{0,t}+\beta_{1,t}WAGE+\beta_{2,t}TRA+\beta_{3,t}EDU+\beta_{4,t}EF+\beta_{5,t}TSRA+\sum \beta_{i,t}Control_{i}+\varepsilon_{\text{t}}$$ (7)

Next, add cultural differences as moderator variables and build the following models with interaction terms:

Model 1a: Employee compensation and international M&A performance with interactive items


$$Y_{t}=\beta_{0,t}+\beta_{1,t}WAGE+\sum \beta_{i,t}Control_{i}+\beta_{8,t}CULD+\beta_{9,t}CULD\times WAGE+\varepsilon_{t}$$ (8)

Model 2a: Employee training and international M&A performance with interactive items


$$Y_{t}=\beta_{0,t}+\beta_{1,t}TRA+\sum \beta_{i,t}Control_{i}+\beta_{8,t}CULD+\beta_{9,t}CULD\times TRA+\varepsilon_{t}$$ (9)

Model 3a: Education level and international M&A performance of employees joining interactive items


$$Y_{t}=\beta_{0,t}+\beta_{1,t}EDU+\sum \beta_{i,t}Control_{i}+\beta_{8,t}CULD+\beta_{9,t}CULD\times EDU+\varepsilon_{t}$$ (10)

Model 4a: Employee mobility and international M&A performance with interactive items


$$Y_{t}=\beta_{0,t}+\beta_{1,t}EF+\sum \beta_{i,t}Control_{i}+\beta_{8,t}CULD+\beta_{9,t}CULD\times EF+\varepsilon_{t}$$ (11)

Model 5a: Technical staff ratio and international M&A performance


$$Y_{t}=\beta_{0,t}+\beta_{1,t}TSRA+\sum \beta_{i,t}Control_{i}+\beta_{8,t}CULD+\beta_{9,t}CULD\times TSRA+\varepsilon_{t}$$ (12)

Model 6a: Corporate Human Capital and International M&A Performance with Interaction Items


$$Y_{t}=\beta_{0,t}+\beta_{1,t}WAGE+\beta_{2,t}TRA+\beta_{3,t}EDU+\beta_{4,t}EF+\beta_{5,t}TSRA+\sum \beta_{i,t}Control_{i}+\beta_{12,t}CULD+$$ (13)

## 4. Empirical analysis

### 4.1. Sample selection and data source

We selected the M&A enterprises from 2008 to 2015 as the research object, and processed the data as follows. Finally, we selected 147 Chinese listed enterprises as the research sample.

(1) Excluding the ST enterprise sample;
(2) Excluding companies with incomplete data disclosure and sample enterprises with abnormal data;
(3) If the same company conducts multiple international mergers and acquisitions in the sample interval, selects the M&A event with the largest transaction amount as the research object.

The variables involved in this paper are: employee salary, employee training investment, employee education level, employee mobility, the proportion of employees and technicians, ROA value of enterprises, and cultural differences. These data come from Wind database, Hofstede index website and enterprise official website, and then are obtained by manual collation.

### 4.2 Descriptive statistical analysis

Using Stata15.0 to describe statistics on the Explained variables, explanatory variables and control variables involved in our paper, we can better understand the various factors of the human capital structure of the enterprise samples, and prepare for the next analysis of the performance of enterprises in international mergers and acquisitions. The descriptive statistics are shown in Table 2:

**Table 2:** Descriptive Statistics of Variables.

variable	Minimum	Maximum	Average	Standard Deviation
Y0	-0.1448	0.1935	0.0561	0.0459
Y1	-0.2246	10.0296	0.1160	0.8246
Y2	-0.2076	0.9424	0.0464	0.0906
Y01	-0.2270	0.1536	-0.0119	0.0406
Y11	-0.3924	9.9102	0.0480	0.8211
Y21	-0.3498	0.8230	-0.0216	0.0972
WAGE	-7.8727	41.7854	5.2356	8.8207
TRA	0.0000	3.7261	0.3205	0.6165
EDU	0.2167	5.0540	1.9730	1.0226
EF	-0.3269	11.4699	0.3271	1.0815
TSRA	0.0000	0.9377	0.2516	0.2221
CULD	0.5688	45.4790	6.5101	4.7848
LNSI	4.9904	13.2069	7.9513	1.4730
AGE	4	33	15.4490	4.7285
EXP	0	1	0.1701	0.3770
INDUS	0	1	0.9388	0.2406
FH	0.0731	0.8635	0.3641	0.1715
GSTYP	0	1	0.1701	0.3770
PIMAP	-0.0838	0.3827	0.0680	0.0562

According to the descriptive statistics, it can be seen that the performance of the current year of M&A relative to the year before M&A and the performance of the second year after M&A relative to the year before M&A are both negative. This shows that the current results of international M&A by Chinese companies are not satisfactory, and there is still much room for improvement in the international M&A market.From the perspective of employees’ educational level, the overall gap is not very large. From the perspective of the value of employee turnover, most of the enterprises engaged in international M&A show an upward trend in the number of employees compared with the previous year due to the occurrence of M&A. In other words, the proportion of personnel inflows in employee turnover is large. From the perspective of the proportion of employees in the company, the proportion of technicians in M&A companies is different and the degree of dispersion is large.

### 4.3 Correlation analysis

Through descriptive statistical tests, we have a certain understanding of the statistical characteristics of the various factors studied. We use SPSS 21.0 to test the correlation between variables. The results are shown in Table 3:

**Table 3:** Practical Correlation Analysis of Company.

Variable	Y0	Y1	Y2	Y01	Y11	Y21	WAGE	TRA	EDU	EF	TSRA	LNSI	AGE	EXP	INDS	FH	GSTYP	PIMAP
Y0	1																	
Y1	.127	1																
Y2	.297^***^	.837^***^	1															
Y01	.160 *	.009	.073	1														
Y11	.079	.998^***^	.828^***^	.049	1													
Y21	-.128	.725^***^	.823^***^	.410^***^	.755^***^	1												
WAGE	.102	-.032	.073	.167^**^	-.029	.090	1											
TRA	.049	.024	.006	-.075	.017	-.049	.127	1										
EDU	.248^***^	.090	.032	.011	.077	-.083	.109	.126	1									
EF	.213^***^	-.019	-.002	-.288^***^	-.046	-.223^***^	.092	.086	-.062	1								
TSRA	.236^***^	.048	.077	.118	.041	.010	-.061	-.064	.605^***^	.021	1							
LNSI	-.048	.028	.036	-.047	.029	.037	-.145 *	-.134	-.175^**^	-.211^***^	-.187^**^	1						
AGE	-.004	-.043	-.007	.014	-.042	.002	.175^**^	-.093	-.036	.128	.012	-.009	1					
EXP	.075	-.035	.038	-.096	-.044	-.040	.121	.114	.003	.107	.114	.180^**^	.152 *	1				
INDUS	-.165^**^	.011	-.107	-.256^***^	.008	-.129	-.171^**^	.038	-.005	-.075	.057	.080	.085	.048	1			
FH	.070	-.035	-.030	-.069	-.043	-.089	-.088	-.114	-.177^**^	.081	-.191^**^	.298^***^	-.024	.125	-.019	1		
GSTYP	-.118	-.038	-.020	.076	-.028	.069	-.027	.048	-.039	-.096	-.070	.365^***^	-.035	.066	-.035	.173^**^	1	
PIMAP	.701^***^	.097	.190^**^	-.592^***^	.029	-.401^***^	-.037	.095	.194^**^	.382^***^	.107	-.005	-.014	.131	.050	.106	-.151 *	1

Note: *  indicates significant at the level of 0.1, ** indicates significant at the level of 0.05, and *** indicates significant at the level of 0.01.

From the results of the correlation test, there is a certain gap between the correlation conclusions and the research hypothesis. First of all, it can be seen that in the short term, employee compensation, employee education, employee turnover, and the proportion of employee technical personnel are all the same as the hypothesis. However, the impact of employee training on the performance of international M&A fails to pass the significance test, which is contrary to the original hypothesis. The reason for the gap may be that the correlation test only considers the relationship between the two variables, and the results may be different when other control variables are introduced. In addition, it can be seen that in the correlation test, the correlation coefficients between explanatory variables are not greater than 0.8. It could be concluded that there was no collinearity between explanatory variables studied in our paper.

### 4.4 Regression analysis

#### 4.4.1. *Data standardization.*

Since the units of each data indicator are different, the data needs to be standardized, which makes the empirical results more accurate. The standardization method used in our paper can be expressed as:


$$x=\frac{X-Minimum}{Maximum-Minimum}$$ (14)

Where x refers to the value after standardization, and X is the original value.

We use SPSS software to directly standardize the data, and perform empirical research on regression analysis based on the standardized data.

#### 4.4.2. Regression Analysis of Enterprise Human Capital Structure and Corporate International M&A Performance.

After ensuring the stability of the variables and the regression method used, the relationship between the human capital structure and the international M&A performance of the enterprise was analyzed by Stata15.0. The results are shown in the following tables:

(1) Employee compensation and international M&A performance

According to the Table 4, from the performance differences between the current year of M&A and the year before the M&A, the employee compensation of the company has a positive impact on the performance of international M&A, and passes the significance test. In the first year and the second year after the completion of the M&A, the impact of employee compensation on international M&A performance is not important. This may be due to the rigidity of employee compensation. the existence of wage rigidity will weaken its incentive effect on employees, so that there is no significant positive relationship between employee compensation and international M&A performance. In addition, it may also be that companies are not able to respond to changes in employee compensation in international M&A, and this also makes the current average salary of employees unable to meet the incentives required by companies in international M&A, so that employee compensation cannot truly realize its positive effect on the performance of international M&A.

**Table 4:** Regression Results of Employee Compensation and International M&A Performance.

Variable	Y0	Y1	Y2	Y01	Y11	Y21
WAGE	0.0745 *	-0.0056	0.0298	0.0662 *	-0.0056	0.0292
LNSI	-0.0079	0.0287	0.0348	-0.007	0.0286	0.0341
AGE	0.0058	-0.0172	-0.004	0.0052	-0.0171	-0.004
EXP	-0.0071	-0.0092	0.001	-0.0063	-0.0091	0.001
INDUS	-0.1033***	0.0006	-0.037	-0.0918***	0.0006	-0.0363
FH	0.0037	-0.0206	-0.0257	0.0033	-0.0205	-0.0252
GSTYP	-0.0033	-0.0075	-0.0017	-0.0029	-0.0075	-0.0017
PIMAP	0.804***	0.0686	0.1332**	-0.511***	0.023	-0.2671***
_cons	0.4104***	0.0188	0.203	0.801***	0.0432	0.3917***
R-squared	0.541	0.0186	0.061	0.4146	0.0101	0.1829
F	20.33***	0.33	1.12	12.22***	0.18	3.86***

Note: *  indicates significant at the level of 0.1, ** indicates significant at the level of 0.05, and *** indicates significant at the level of 0.01.

(2) Staff training investment and international M&A performance

It can be seen from the Table 5 that whether it is the year of merger, the first year after merger or the second year after merger, the impact of employee training investment on the performance of international M&A has not passed the significance test.In other words, employee training has no significant impact on the performance of enterprises’ international M&A, so the above hypothesis 2 fail to be verified. The reason for this result may be that, firstly, the “employee education funds” in the annual report of the enterprise may be the strategic behavior of the enterprise tax avoidance. In fact, the employees are not trained to make the return results untrue. Secondly, the investment in employee training will not pay off for a year or two. It takes a long period of time from the training input to its function, and the output cannot be achieved in a short period of time. Therefore, during the time period studied in our paper, the impact of employee training on the performance of international M&A may not be fully demonstrated. Thirdly, the current staff training system is not perfect enough to really play its role, which means that the impact of the corporate training system on the performance of international M&A is not enough, so it is difficult to exert its incentive effect.

**Table 5:** Regression Results of Staff Training Investment and International M&A Performance.

Variable	Y0	Y1	Y2	Y01	Y11	Y21
TRA	-0.0092	0.0106	-0.0045	-0.0082	0.0105	-0.0044
LNSI	-0.0201	0.0313	0.0298	-0.0178	0.0312	0.0292
AGE	0.0182	-0.0169	0.0008	0.0161	-0.0168	0.0008
EXP	-0.0017	-0.0102	0.0032	-0.0015	-0.0102	0.0032
INDUS	-0.1128***	0.0009	-0.0408	-0.1002***	0.0009	-0.04
FH	-0.0008	-0.0194	-0.0276	-0.0007	-0.0193	-0.0271
GSTYP	-0.002	-0.0082	-0.0012	-0.0018	-0.0082	-0.0012
PIMAP	0.8014***	0.0674	0.1323**	-0.5133***	0.0218	-0.268***
_cons	0.4406***	0.0153	0.2152	0.8279***	0.0397	0.4037***
R-squared	0.5326	0.0189	0.057	0.4038	0.0104	0.1795
F	19.66**	0.33	1.04	11.68***	0.18	3.77***

Note: *  indicates significant at the level of 0.1, ** indicates significant at the level of 0.05, and *** indicates significant at the level of 0.01.

(3) Employee education level and international M&A performance

According to the Table 6, from the performance of the year of the international M&A and the year before the international M&A, it can be seen that the education level of employees is significantly positively correlated with the performance of international M&A. The first year after the M&A is not significant, the reason for this phenomenon may be that after the international M&A stabilized, other important factors affecting international M&A began to gradually play its leading role, such as the innovation ability of enterprises, product performance, etc. However, the effect of the employee’s education level on the performance will be gradually weaken, and it will no longer significantly affect the performance of international M&A.

**Table 6:** Regression Results of Employee Education Level and International M&A Performance.

Variable	Y0	Y1	Y2	Y01	Y11	Y21
EDU	0.0780 *	0.0306	-0.004	0.0692 *	0.0305	-0.0039
LNSI	-0.0048	0.0349	0.0298	-0. 0043	0.0348	0.0292
AGE	0.0231	-0.0167	0.0012	0.0205	-0.0166	0.0012
EXP	-0.0033	-0.001	0.003	-0.0029	-0.0098	0.0029
INDUS	-0.1130***	0.0014	-0.041	-0.1004***	0.0013	-0.0402
FH	0.0125	-0.0154	-0.0279	0.0111	-0.0153	-0.0273
GSTYP	-0.0058	-0.0089	-0.0013	-0.0052	-0.0088	-0.0013
PIMAP	0.7713***	0.0576	0.1332**	-0.5400***	0.0123	-0.2672***
_cons	0. 4114***	0.0056	0.2161***	0.8018***	0.023	0.4046
R-squared	0.5447	0.0238	0.057	0.0867	0.0153	0.1795
F	20.64***	0.42	1.04	12.45***	0.27	3.77***

Note: *  indicates significant at the level of 0.1, ** indicates significant at the level of 0.05, and *** indicates significant at the level of 0.01.

(4) Employee turnover and international M&A performance

According to the Table 7, from the performance difference between the current year of the M&A and the year before the M&A, the negative correlation between employee turnover and international M&A performance has passed the significance test. However, after the completion of the first year of M&A, the negative impact of employee turnover on international performance was not significant. The reason for this phenomenon may be that, firstly, the retired employees have been effectively replaced, and the new inflows are skilled in the business, making the negative impact of employee turnover on performance be weakened. Secondly, after the completion of the M&A, the employee flow tends to be stable, and other important factors affecting international M&A begin to play a leading role.

**Table 7:** Regression Results of Employee Turnover and International M&A Performance.

Variable	Y0	Y1	Y2	Y01	Y11	Y21
EF	-0.158 *	-0.0389	-0.0789	-0.1404 *	-0.0387	-0.0774
LNSI	-0.0393	0.0244	0.0201	-0.035	0.0243	0.0197
AGE	0.0308	0.0154	0.0071	0.0274	-0.0153	0.007
EXP	0.0004	0.0088	0.0043	0.0004	-0.0088	0.0042
INDUS	-0.1182***	0	-0.0435	-0.1051***	0	-0.0427
FH	0.007	-0.0186	0.0237	0.0062	-0.0185	-0.0233
GSTYP	-0.0013	-0.0073	-0.0008	-0.0012	-0.0072	-0.0008
PIMAP	0.8448***	0.0799	0.154	-0.4748***	0.0342	-0.2467
_cons	0.4382***	0.0163	0.214	0.8257***	0.0407	0.4025
R-squared	0.5413	0.02	0.0634	0.4148	0.0115	0.185
F	20.35***	0.35	1.17	12.23***	0.2	3.92***

Note: *  indicates significant at the level of 0.1, ** indicates significant at the level of 0.05, and *** indicates significant at the level of 0.01.

(5) Technical personnel ratio and international M&A performance

According to the Table 8, from the performance of the M&A year and the difference between the performance of the M&A year and the performance of the year before the M&A,it can be seen that the proportion of enterprise technicians is positively related to international M&A, and passed the significance test. After the first year of M&A, the positive impact of the proportion of technicians on international M&A performance did not pass the significance test. This may be because the ability of technicians was not well integrated during the M&A process, so that the synergy effect after M&A is not well played.

**Table 8:** Regression Results of Technical Personnel Ratio and International M&A Performance.

Variable	Y0	Y1	Y2	Y01	Y11	Y21
TSRA	0.1054***	0.0156	0.0217	0.0937***	0.0155	0.0213
LNSI	0.0064	0.0332	0.0357	0.0056	0.0331	0.0349
AGE	0.0222	-0.0178	0.002	0.0197	-0.0177	0.002
EXP	-0.012	-0.011	0.0009	-0.0107	-0.0109	0.0009
INDUS	-0.1192***	0.0004	-0.0422	-0.1059***	0.0004	-0.0414
FH	0.02	-0.0174	-0.0231	0.0177	-0.0173	-0.0227
GSTYP	-0.0048	-0.0079	-0.0019	-0.0042	-0.0079	-0.0019
PIMAP	0.778***	0.0656	0.1271**	-0.5341***	0.02	-0.2731***
_cons	0.4088***	0.0121	0.2083***	0.7995***	0.0364	0.397***
R-squared	0.5632	0.0204	0.0608	0.4428	0.0119	0.1828
F	22.24***	0.36	1.12	13.71***	0.21	3.86***

Note: *  indicates significant at the level of 0.1, ** indicates significant at the level of 0.05, and *** indicates significant at the level of 0.01.

(6) Corporate human capital and international M&A performance

According to the Table 9, from the difference between the year of the M&A and the year of the M&A relative to the year before the M&A, it can be seen that employee compensation is significantly positively correlated with international M&A performance. The employee turnover is negatively correlated with the performance of international M&A and passed the significance test. The proportion of technical personnel is significantly positively correlated with international M&A performance. Whether in the first year or the second year after the completion of the merger or acquisition, the relationship among employee training, employee education level and international M&A performance has not passed the significance test.

**Table 9:** Regression Results of Human Capital and International M&A Performance.

Variable	Y0	Y1	Y2	Y01	Y11	Y21
WAGE	0.0985**	-0.0091	0.0434	0.0875**	-0.009	0.0425
TRA	0.0113	0.0106	0.0059	0.01	0.0106	0.0057
EDU	-0.0287	0.0275	-0.0477	-0.0255	0.0274	-0.0468
EF	-0.1585 *	-0.0264	-0.0962	-0.1409 *	-0.0262	-0.0942
TSRA	0.1259***	0.0018	0.0476	0.1119***	0.0017	0.0466
LNSI	0.0012	0.0318	0.0279	0.0011	0.0316	0.0273
AGE	0.0165	-0.0119	0.0003	0.0147	-0.0119	0.0002
EXP	-0.0179	-0.0097	-0.0023	-0.0159	-0.0096	-0.0022
INDUS	-0.1129***	-0.0012	-0.0413	-0.1003***	-0.0011	-0.0405
FH	0.0322	-0.0139	-0.0189	0.0286	-0.0138	-0.0185
GSTYP	-0.0044	-0.0091	-0.0004	-0.0039	-0.009	-0.0004
PIMAP	0.8327***	0.0638	0.168***	-0.4854***	0.0182	-0.233***
_cons	0.372***	0.0081	0.1995***	0.7668***	0.0325	0.3883***
R-squared	0.5846	0.0252	0.0802	0.4701	0.0167	0.1996
F	15.71***	0.29	0.97	9.91***	0.19	2.79***

Note: *  indicates significant at the level of 0.1, ** indicates significant at the level of 0.05, and *** indicates significant at the level of 0.01.

#### 4.4.3. Analysis of the adjustment role of cultural differences.

Next, we introduced cultural differences as an interaction term, and used Stata 15.0 to conduct regression analysis on the moderating effects of cultural differences.The analysis results are shown in the following table.

According to the Table 10,from the difference between the second year after the M&A and the second year after the M&A relative to the year before the M&A,it can be seen that the regression coefficient of employee compensation and international M&A performance is positive, while the interaction coefficient of employee compensation and cultural difference is negative, and both have passed the significance test.This result indicated that cultural differences weaken the significant positive influence of employee compensation on the performance of international M&A.

**Table 10:** Regression Results of the Cultural Differences Adjustment on the Relationship between Employee Compensation and International M&A Performance.

Variable	Y0	Y1	Y2	Y01	Y11	Y21
WAGE	0.094	0.0968	0.1267 *	0.0835	0.0963	0.1242 *
CULD•WAGE	-0.1753	-0.9155	-0.8657 *	-0.1558	-0.9111	-0.8489 *
CULD	0.1404	0.3876**	0.3549**	0.1248	0.3858**	0.348**
LNSI	0.0198	0.0009	0.0097	-0.0175	0.0009	0.0094
AGE	0.013	-0.0022	0.0093	0.0116	-0.0022	0.0091
EXP	-0.0092	-0.0161	-0.0054	-0.0082	-0.016	-0.00532
INDUS	-0.1067***	0.002	-0.035	-0.0948***	0.002	-0.0343
FH	0.0017	-0.0202	-0.025	0.0014	-0.0201	-0.0244
GSTYP	-0.0005	-0.0042	0.0011	-0.0004	-0.0041	0.001
PIMAP	0.8105***	0.0755	0.1389**	-0.5052***	0.0299	-0.2615***
_cons	0.3958***	-0.0274	0.1602***	0.788***	-0.0028	0.3498***
R-squared	0.5467	0.0789	0.1113	0.4218	0.071	0.2267
F	16.40***	1.17	1.70 *	9.92***	1.04	3.99***

Note: *  indicates significant at the level of 0.1, ** indicates significant at the level of 0.05, and *** indicates significant at the level of 0.01.

It can be seen from the Table 11 that after the introduction of cultural differences, both in the short-term and in the long-term, neither the training input nor the interaction between the training input and the cultural difference can pass the significance test, indicating that cultural differences have no obvious moderating effect on the relationship between employee training and international M&A performance.

**Table 11:** Regression Results of the Cultural Differences Adjustment on the Relationship between Staff Training and International M&A Performance.

Variable	Y0	Y1	Y2	Y01	Y11	Y21
TRA	0.0111	-0.0763	-0.0998	0.0098	-0.0759	-0.0978
CULD•TRA	-0.1661	0.6721	0.7394	-0.1476	0.669	0.7251
CULD	0.1085	0.1191	0.0947	0.0964	0.1185	0.0929
LNSI	-0.0301	0.0155	0.016	-0.0267	0.0154	0.0157
AGE	0.0241	-0.0046	0.012	0.0214	-0.0045	0.0117
EXP	-0.0035	-0.0093	0.0046	-0.0031	-0.0092	0.0045
INDUS	-0.1178***	-0.0093	-0.0501 *	-0.1047***	-0.0093	-0.0491 *
FH	-0.0051	-0.0203	-0.0274	-0.0045	-0.0202	-0.0269
GSTYP	0.0017	-0.0049	0.0011	0.0015	-0.0049	0.0011
PIMAP	0.8105***	0.0745	0.1373**	-0.5052***	0.0289	-0.263***
_cons	0.4307***	0.0072	0.2094***	0.8191***	0.0316	0.3981***
R-squared	0.5383	0.0725	0.1048	0.4111	0.0645	0.221
F	15.86***	1.06	1.59	9.50***	0.94	3.86***

Note: *  indicates significant at the level of 0.1, ** indicates significant at the level of 0.05, and *** indicates significant at the level of 0.01.

From the Table 12, it can be seen from the performance differences between the current year of M&A and the year before the M&A, the regression coefficient of employee education level and international M&A performance is positive, and the significance test is passed. The coefficient of interaction between the difference and the education level of employees did not pass the significance test, indicating that cultural differences have no adjustment effect on the positive impact of employee education level and international M&A performance. In the first year and the second year after the M&A, the regression coefficient of the education level of the employees and the international M&A performance were both negative and significant at the level of 0.01. the interaction coefficient between cultural difference and employees’ education level was positive, and all passed the significance test, indicating that cultural differences would reduce the negative impact of education on international M&A performance. This may be because cultural differences have a positive impact on the M&A companies. Cultural differences will bring learning effects and complementary effects to both companies, thus reducing the negative impact of education on international M&A performance.

**Table 12:** Regression Results of the Cultural Differences Adjustment on the Relationship between Staff Education Level and International M&A Performance.

Variable	Y0	Y1	Y2	Y01	Y11	Y21
EDU	0.1646**	-0.1905***	-0.1807***	0.1464**	-0.1896***	-0.1772***
CULD•EDU	-0.6168	2.0784***	1.662***	-0.5483	2.0686***	1.6297***
CULD	0.2492**	-0.2057**	-0.1634 *	0.2215**	-0.2047**	-0.1602 *
LNSI	-0.0198	0.0348	0.0296	-0.0176	0.0346	0.029
AGE	0.0377	-0.0204	-0.0016	0.0335	-0.0203	-0.0016
EXP	-0.0111	0.0075	0.0169	-0.0098	0.0075	0.0166
INDUS	-0.1165***	-0.0217	-0.0595**	-0.1036***	-0.0216	-0.0584**
FH	0.0104	-0.0176	-0.0296	0.0092	-0.0175	-0.029
GSTYP	-0.0029	-0.0009	0.005	-0.0026	-0.0009	0.005
PIMAP	0.7693***	0.0869 *	0.1566***	-0.5417***	0.0412	-0.2441***
_cons	0.3786***	0.0347	0.2392***	0.7728***	0.059	0.4273***
R-squared	0.5613	0.2932	0.2368	0.4404	0.287	0.3359
F	17.4***	5.64***	4.22***	10.7***	5.48***	6.88***

Note: *  indicates significant at the level of 0.1, ** indicates significant at the level of 0.05, and *** indicates significant at the level of 0.01.

As can be seen from the Table 13, as with employee training, after introducing cultural differences, both the short-term and the long-term, the regression coefficients of both employee turnover and interaction between employee turnover and cultural differences have not been able to pass significance test.The results show that cultural differences have no significant regulatory effect on the relationship between employee turnover and international M&A performance.

**Table 13:** Regression Results of the Cultural Differences Adjustment on the Relationship between Employee Turnover and International M&A Performance.

Variable	Y0	Y1	Y2	Y01	Y11	Y21
EF	-0.324	0.2036	0.0467	-0.2879	0.2026	0.04588
CULD•EF	1.8873	-2.8249	-1.4769	1.6773	-2.811	-1.4483
CULD	-0.0113	0.3288**	0.2297 *	-0.01	0.327**	0.2253 *
LNSI	-0.0476	0.0049	0.004	-0.0423	0.0049	0.0039
AGE	0.0339	0.0012	0.0197	0.0301	0.0012	0.0193
EXP	-0.0027	-0.0078	0.0041	0.0024	-0.0077	0.004
INDUS	-0.1277***	-0.0032	-0.0485 *	-0.1135	-0.0032	-0.0476 *
FH	0	-0.0179	-0.0252	0	-0.0178	-0.0247
GSTYP	0.0017	-0.0016	0.0039	0.0015	-0.0016	0.0039
PIMAP	0.8566***	0.088	0.163***	-0.4642	0.0423	-0.2378***
_cons	0.4455***	-0.0219	0.1889***	0.8322	0.0025	0.3779***
R-squared	0.5494	0.0787	0.1026	0.4252	0.0708	0.2191
F	16.58***	1.16	1.55	10.06***	1.04	3.82***

Note: *  indicates significant at the level of 0.1, ** indicates significant at the level of 0.05, and *** indicates significant at the level of 0.01.

It can be seen from the Table 14 that in the year of M&A, the regression coefficient of employee technical personnel and international M&A performance is positive, and the significance test is passed. While the coefficient of interaction between cultural difference and technical personnel is negative. It does not pass the significance test, which shows that cultural differences have not weakened the positive impact of the proportion of employees in the international M&A performance. It can be seen from the first year and the second year after the M&A that the regression coefficient of the ratio of employee technicians to international M&A is negative and has passed the significance test. While the coefficient of interaction between cultural differences and technical personnel is a positive value, and passes the significance test, indicating that cultural differences can reduce the negative impact of technicians on international M&A performance. This is because the existence of cultural differences makes the M&A companies have different technical levels. The companies on both sides of the M&A benefit from this and increase the internalization of proprietary technology, thereby reducing the negative impact of the proportion of technicians on international M&A performance.

**Table 14:** Regression Results of the Cultural Differences Adjustment on the Relationship between Human Capital and International M&A Performance.

Variable	Y0	Y1	Y2	Y01	Y11	Y21
TSRA	0.1617**	-0.2061***	-0.1407***	0.1437**	-0.2051***	-0.138***
CULD•TSRA	-0.4609	2.3613***	1.751***	-0.4096	2.3502***	1.717***
CULD	0.1927**	-0.1	-0.0482	0.1712**	0.0996	-0.0473
LNSI	0.0051	0.019	0.0229	-0.0045	0.0189	0.0225
AGE	0.0342	-0.018	0.0036	0.0303	-0.0179	0.0035
EXP	-0.017	-0.0028	0.0064	-0.0151	-0.0028	0.0063
INDUS	-0.1254***	-0.0206	-0.0593**	-0.1115***	-0.0204	-0.0582**
FH	0.0172	-0.0194	-0.025	0.0152	-0.0192	-0.0245
GSTYP	-0.002	0.0055	0.0088	-0.0018	0.0054	0.0086
PIMAP	0.7844***	0.0873 *	0.1448***	-0.5283***	0.0416	-0.2557***
_cons	0.387***	0.0268	0.2163***	0.7802***	0.0511	0.4048***
R-squared	0.5765	0.2517	0.2053	0.4598	0.2452	0.3085
F	18.51***	4.57***	3.51***	11.58***	4.42***	6.07***

Note: *  indicates significant at the level of 0.1, ** indicates significant at the level of 0.05, and *** indicates significant at the level of 0.01.

As can be seen from the Table 15, in the year of M&A, the impact of various factors of human capital on international M&A performance has not passed the significance test. From the first year and the second year after M&A, the regression coefficient of employee compensation for international M&A performance is positive and passed the significance test, while the coefficient of interaction between cultural difference and employee compensation is negative, and the significance test is also passed, which shows the cultural difference can reduce the positive impact of employee compensation on international M&A performance. The coefficient of education of employees is negative and significant. The regression coefficients of cultural differences and the educational items of employees are positive, and the significance test is passed, indicating that cultural differences can weaken the education level of employees and international M&A. The impact of employee compensation, employee turnover and employee technical staff on international M&A performance is not significant, and their interactions with cultural differences have not passed the significance test.

**Table 15:** Regression Results of the Cultural Differences Adjustment on the Relationship between Human Capital and International M&A Performance.

Variable	Y0	Y1	Y2	Y01	Y11	Y21
WAGE	0.1083	0.1398**	0.1819***	0.0962	0.1392**	0.1784***
CULD•WAGE	-0.0924	-1.3604**	-1.2895**	-0.0821	-1.354**	-1.2645**
TRA	-0.0043	0.0856	0.0404	-0.0038	0.0852	0.0396
CULD•TRA	0.1428	-0.6912	-0.3592	0.1269	-0.6879	-0.3523
EDU	0.0533	-0.2147***	-0.2687***	0.0474	-0.2137***	-0.2635***
CULD•EDU	-0.5514	2.1453***	1.9451***	-0.4901	2.1352***	1.9073***
EF	-0.1259	-0.0887	-0.2347	-0.1119	-0.0883	-0.2301
CULD•EF	-0.2208	0.4159	1.3629	-0.1962	0.4139	1.3364
TSRA	0.1268	-0.0302	0.0495	0.1127	-0.0301	0.0485
CULD•TSRA	-0.0096	0.5177	0.1358	-0.0085	0.5153	0.1331
CULD	0.2705	0.0784	0.0306	0.2404	0.0781	0.03
LNSI	-0.0125	0.0105	0.0103	-0.0111	0.0105	0.01
AGE	0.0319	-0.0143	0.0022	0.0283	-0.0143	-0.0021
EXP	-0.0252	0.0001	0.0052	-0.0224	0.0001	0.0051
INDUS	-0.1156***	-0.013	-0.0516 *	-0.1027***	-0.0129	-0.0506 *
FH	0.0319	-0.0115	-0.0171	0.0284	-0.0114	-0.0167
GSTYP	-0.0022	0.0012	0.0062	-0.002	0.0012	0.0061
PIMAP	0.8245***	0.1045 *	0.2009***	-0.4927***	0.0587	-0.2006***
_cons	0.3353***	-0.0021	0.1978***	0.7343***	0.0222	0.3867***
R-squared	0.6001	0.3554	0.3107	0.4899	0.3498	0.4002
F	10.67***	3.92***	3.20***	6.83***	3.83***	4.74***

Note: *  indicates significant at the level of 0.1, ** indicates significant at the level of 0.05, and *** indicates significant at the level of 0.01.

### 4.5. Robustness test

To verify that the above empirical results are reliable, we conducted a robustness test. We chooses to use the return on equity (ROE) to replace the international M&A performance and shorten the sample selection time to conduct the robustness test.Since the number of listed companies with international M&A events in 2008-2010 is very small, we select listed companies with international M&A events in 2011-2015 for regression analysis, which has reached the robustness test. The regression data was analyzed by the measurement software Stata15.0. The test results are shown in the following table:

#### 4.5.1. Replacing the metric for the dependent variable.

According to the test results in the Tables 16–21, the significance of each influencing factor is basically consistent with the previous test results. Although the results of some control variables are inconsistent with the results listed above, which do not affect the overall test results. This further proves the validity and stability of the empirical analysis results.

**Table 16:** Regression Results of Employee Compensation and International M&A Performance.

Variable	Y0	Y1	Y2	Y01	Y11	Y21
WAGE	0.0865**	0.0090	0.0137	0.1160**	0.0089	0.0014
LNSI	0.0154	-0.0321	0.0178	0.0206	-0.0388	0.0131
AGE	0.0286	0.0226	0.0751	0.0383	0.0273	0.0553
EXP	0.0072	0.0050	0.0289	0.0096	0.0060	0.0212
INDUS	’-0.0544 *	-0.0252	-0.0736 *	-0.0730 *	-0.0305	-0.0542 *
FH	0.0301	0.0329	0.0221	0.0404	0.0397	0.0163
GSTYP	-0.0084	0.0138	0.0155	-0.0113	0.0167	0.0114
PIMAP	0.6028***	0.2956***	0.2524***	-0.5662***	-0.4213***	-0.4712***
_cons	0.3517***	0.5170***	0.4863***	0.7607***	0.7878***	0.6218***
R-squared	0.5319	0.2283	0.1929	0.3758	0.3040	0.4497
F	19.60***	5.10***	4.12***	10.38***	7.54***	14.10***

Note: *  indicates significant at the level of 0.1, ** indicates significant at the level of 0.05, and *** indicates significant at the level of 0.01

**Table 17:** Regression Results of Staff Training and International M&A Performance.

Variable	Y0	Y1	Y2	Y01	Y11	Y21
TRA	0.0052	0.0227	-0.0141	0.0070	0.0275	-0.0104
LNSI	0.0044	-0.0401	-0.0042	0.0059	-0.0484	-0.0031
AGE	0.0450	0.0419	0.0984 *	0.0604	0.0506	0.0724 *
EXP	0.0122	0.0091	0.0384	0.0164	0.0110	0.0283
INDUS	-0.066**	-0.0378	-0.0913**	-0.0885**	-0.0456	-0.0672**
FH	0.0262	0.0306	0.0136	0.0352	0.0369	0.0101
GSTYP	-0.0077	0.0135	0.0180	-0.0104	0.0163	0.0133
PIMAP	0.6003***	0.2902***	0.2518***	-0.5696***	-0.4279***	-0.4716***
_cons	0.3837***	0.5481***	0.5404***	0.8037***	0.8253***	0.6616***
R-squared	0.5166	0.2029	0.1551	0.3553	0.2811	0.4239
F	18.43***	4.3900	3.17***	9.51***	6.74***	12.69***

**Table 18:** Regression Results of Staff Education Level and International M&A Performance.

Variable	Y0	Y1	Y2	Y01	Y11	Y21
EDU	0.0482 *	0.0502	0.0673	0.0647 *	0.0605	-0.0496
LNSI	0.0164	-0.0311	-0.0196	0.0220	-0.0375	-0.0144
AGE	0.0473	0.0421	0.0960 *	0.0634	0.0508	0.0707 *
EXP	0.0118	0.0098	0.0386	0.0158	0.0118	0.0284
INDUS	-0.0645**	-0.0358	-0.0936**	-0.0866**	-0.0430	-0.0689**
FH	0.0352	0.0382	0.0017	0.0473	0.0461	0.0012
GSTYP	-0.0096	0.0126	0.0202	-0.0129	0.0151	0.0149
PIMAP	0.5784***	0.2702***	0.2812***	-0.5989***	-0.4519***	-0.4450***
_cons	0.3661***	0.5321***	0.5641***	0.7780***	0.8060***	0.6791***
R-squared	0.5225	0.2118	0.1662	0.3632	0.2891	0.4314
F	18.88***	4.63***	3.44***	9.84***	7.02***	13.09***

Note: *  indicates significant at the level of 0.1, ** indicates significant at the level of 0.05, and *** indicates significant at the level of 0.01

**Table 19:** Regression Results of Employee Turnover and International M&A Performance.

Variable	Y0	Y1	Y2	Y01	Y11	Y21
EF	0.0357	-0.1115	0.2068	0.0479	-0.1347	0.1522
LNSI	0.0093	-0.0632	0.0331	0.0124	-0.0763	0.0243
AGE	0.0420	0.0461	0.0869	0.0564	0.0557	0.0640
EXP	0.0117	0.0134	0.0323	0.0157	0.0162	0.0238
INDUS	-0.0644**	-0.0414	-0.0839**	-0.0864**	-0.0499	-0.0617**
FH	0.0242	0.0328	0.0066	0.0325	0.0396	0.0049
GSTYP	-0.0074	0.0150	0.0168	-0.0100	0.0181	0.0124
PIMAP	0.5949***	0.3129***	0.2142***	-0.5767***	-0.4004***	-0.4992***
_cons	0.3829***	0.5559***	0.5296***	0.8026***	0.8347***	0.6537***
R-squared	0.5172	0.2117	0.1763	0.3561	0.289	0.4384
F	18.48***	4.63***	3.69***	9.54***	7.01***	13.46***

Note: *  indicates significant at the level of 0.1, ** indicates significant at the level of 0.05, and *** indicates significant at the level of 0.01

**Table 20:** Regression Results of Technical Staff Ratio and International M&A Performance.

Variable	Y0	Y1	Y2	Y01	Y11	Y21
TSRA	0.0662**	0.0386	0.0207	0.0888**	0.0466	0.0152
LNSI	0.0211	-0.0343	0.0041	0.0283	-0.0414	0.0031
AGE	0.0466	0.0403	0.1009 *	0.0626	0.0487	0.0743 *
EXP	0.0061	0.0069	0.0354902	0.0083	0.0083	0.0261
INDUS	-0.0692**	0.0391	-0.0928**	-0.0928**	-0.0472	-0.0683**
FH	0.0387	0.0358	0.0191	0.0519	0.0433	0.0141
GSTYP	-0.0085	-0.0142	0.0167	-0.0114	0.0171	0.0123
PIMAP	0.5905***	0.2877***	0.2463***	-0.5826***	-0.4309***	-0.4756***
_cons	0.3631***	0.5387***	0.5317***	0.7759***	0.8139***	0.6553
R-squared	0.5328	0.2103	0.1563	0.3769	0.2877	0.4247
F	19.67***	4.59***	3.20***	10.43***	6.97***	12.74***

Note: *  indicates significant at the level of 0.1, ** indicates significant at the level of 0.05, and *** indicates significant at the level of 0.01

**Table 21:** Regression Results of Human Capital and International M&A Performance.

Variable	Y0	Y1	Y2	Y01	Y11	Y21
WAGE	0.1002 **	0.0969 **	0.1686 ***	0.1343**	0.1170 **	0.1241 ***
TRA	0.0118	0.0244	-0.0072	0.0158	0.0295	-0.0053
EDU	-0.0189	-0.0025	-0.1459 **	-0.0253	-0.0031	-0.1074**
EF	0.0306	-0.1150	0.1537	0.0410	-0.1388	0.1131
TSRA	0.0859 **	0.0493	0.1082 **	0.1152 **	0.0595	0.0796***
LNSI	0.0426	-0.0328	0.0357	0.0572	-0.0397	0.0263
AGE	0.0274	0.0333	0.0535	0.0368	0.0402	0.0393
EXP	-0.0032	0.0010	0.0154	-0.0044	0.0012	0.0113
INDUS	-0.0566 *	-0.0319	-0.0727 *	-0.0759 *	-0.0386	-0.0535 *
FH	0.0438	0.0495	0.0090	0.0588	0.0597	0.0066
GSTYP	-0.0099	0.0116	0.0202	-0.0133	0.0140	0.0149
PIMAP	0.5912 ***	0.3049 ***	0.2791 ***	-0.5817 ***	-0.4101 ***	-0.4514 ***
_cons	0.3230 ***	0.5010 ***	0.4896***	0.7222 ***	0.7684***	0.6243 ***
R-squared	0.5538	0.2535	0.2505	0.4049	0.3267	0.4889
F	13.86***	3.79***	3.73***	7.60***	5.42***	10.68***

Note: *  indicates significant at the level of 0.1, ** indicates significant at the level of 0.05, and *** indicates significant at the level of 0.01

#### 4.5.2. Shortening the sample selection time.

According to the test results in the Tables 22–26, the test results of the impact of employee compensation, employee training investment, employee education level, employee fluidity and technical staff ratio on the performance of international M&A performance are basically consistent with the previous test results, and the significance of each influencing factor is basically consistent with the previous test results. Once again, it proves the effectiveness and stability of the empirical analysis results in this paper.

**Table 22:** Regression Results of Employee Compensation and International M&A Performance.

Variable	Y0	Y1	Y2	Y01	Y11	Y21
WAGE	0.079 *	0.004 *	0.038 *	0.070 *	0.004 *	0.037 *
LNSI	-0.032	-0.001	0.007	-0.028	-0.001	0.007
AGE	0.003	-0.001	0.017	0.003	-0.001	0.016
EXP	-0.008	0.000	0.010	-0.007	0.000	0.010
INDUS	-0.116***	-0.003 *	-0.038**	-0.103***	-0.003 *	-0.037**
FH	-0.002	0.001	-0.002	-0.002	0.001	-0.002
GSTYP	-0.012	0.000	0.003	-0.011	0.000	0.003
PIMAP	0.800***	0.015***	0.078**	-0.515***	-0.031***	-0.321***
_cons	0.445***	0.023***	0.207***	0.832***	0.047***	0.396***
R-squared	0.564	0.226	0.148	0.459	0.510	0.501
F	13.567	3.064	1.828	8.922	10.926	10.545

Note: *  indicates significant at the level of 0.1, ** indicates significant at the level of 0.05, and *** indicates significant at the level of 0.01

**Table 23:** Regression Results of Staff Training and International M&A Performance.

Variable	Y0	Y1	Y2	Y01	Y11	Y21
TRA	-0.022	-0.002	-0.032	-0.019	-0.002	-0.032
LNSI	-0.046	-0.002	-0.003	-0.041	-0.002	-0.003
AGE	0.017	0.000	0.022	0.015	0.000	0.022
EXP	-0.002	0.001	0.014	-0.002	0.001	0.014
INDUS	-0.126^***^	-0.004^**^	-0.042^***^	-0.112^***^	-0.004^**^	-0.042^***^
FH	-0.008	0.000	-0.006	-0.007	0.000	-0.006
GSTYP	-0.009	0.000	0.006	-0.008	0.000	0.006
PIMAP	0.795^***^	0.014^***^	0.076^**^	-0.519^***^	-0.031^***^	-0.324^***^
_cons	0.482^***^	0.025^***^	0.229^***^	0.865^***^	0.049^***^	0.417^***^
R-squared	0.554	0.207	0.139	0.448	0.498	0.495
F	13.063	2.740	1.690	8.515	10.414	10.309

Note: *  indicates significant at the level of 0.1, ** indicates significant at the level of 0.05, and *** indicates significant at the level of 0.01

**Table 24:** Regression Results of Staff Education Level and International M&A Performance.

Variable	Y0	Y1	Y2	Y01	Y11	Y21
EDU	0.084^**^	0.003^ * ^	-0.025	0.075^**^	0.003^ * ^	-0.025
LNSI	-0.026	-0.001	-0.003	-0.024	-0.001	-0.003
AGE	0.024	0.000	0.022	0.021	0.000	0.022
EXP	-0.003	0.000	0.012	-0.003	0.000	0.012
INDUS	-0.124^***^	-0.003^**^	-0.044^***^	-0.110^***^	-0.003^**^	-0.043^***^
FH	0.006	0.001	-0.008	0.005	0.001	-0.008
GSTYP	-0.016	0.000	0.006	-0.014	0.000	0.006
PIMAP	0.771^***^	0.013^***^	0.083^**^	-0.541^***^	-0.032^***^	-0.316^***^
_cons	0.442^***^	0.023^***^	0.234^***^	0.829^***^	0.047^***^	0.422^***^
R-squared	0.567	0.223	0.139	0.464	0.508	0.496
F	13.773	3.018	1.694	9.088	10.854	10.316

Note: *  indicates significant at the level of 0.1, ** indicates significant at the level of 0.05, and *** indicates significant at the level of 0.01

**Table 25:** Regression Results of Employee Turnover and International M&A Performance.

Variable	Y0	Y1	Y2	Y01	Y11	Y21
EF	-0.174^ * ^	-0.014^***^	-0.056	-0.154^ * ^	-0.014^***^	-0.055
LNSI	-0.066	-0.004	-0.006	-0.058	-0.004	-0.006
AGE	0.030	0.001	0.028	0.027	0.001	0.027
EXP	-0.001	0.001	0.012	-0.001	0.001	0.012
INDUS	-0.132^***^	-0.004^***^	-0.045^***^	-0.118^***^	-0.004^***^	-0.044^***^
FH	-0.001	0.001	-0.003	-0.001	0.001	-0.003
GSTYP	-0.008	0.000	0.005	-0.007	0.000	0.005
PIMAP	0.847^***^	0.019^***^	0.093^**^	-0.473^***^	-0.027^***^	-0.307^***^
_cons	0.472^***^	0.024^***^	0.221^***^	0.856^***^	0.048^***^	0.409^***^
R-squared	0.565	0.269	0.138	0.461	0.537	0.495
F	13.629	3.865	1.685	8.972	12.191	10.302

Note: *  indicates significant at the level of 0.1, ** indicates significant at the level of 0.05, and *** indicates significant at the level of 0.01

**Table 26:** Regression Results of Technical Staff Ratio and International M&A Performance.

Variable	Y0	Y1	Y2	Y01	Y11	Y21
TSRA	0.110^***^	0.018	0.003^**^	0.097^***^	0.003^**^	0.017
LNSI	-0.013	0.007	-0.001	-0.011	-0.001	0.007
AGE	0.030	0.026	0.000	0.026	0.000	0.025
EXP	-0.013	0.010	0.000	-0.011	0.000	0.010
INDUS	-0.131^***^	-0.044^***^	-0.004^**^	-0.117^***^	-0.004^**^	-0.043^***^
FH	0.014	-0.001	0.001	0.012	0.001	-0.001
GSTYP	-0.015	0.003	0.000	-0.014	0.000	0.003
PIMAP	0.772^***^	0.072^**^	0.014^***^	-0.539^***^	-0.032^***^	-0.327^***^
_cons	0.452^***^	0.219^***^	0.024^***^	0.838^***^	0.048^***^	0.407^***^
R-squared	0.585	0.136	0.232	0.486	0.514	0.494
F	14.810	1.648	3.170	9.925	11.093	10.239

Note: *  indicates significant at the level of 0.1, ** indicates significant at the level of 0.05, and *** indicates significant at the level of 0.01

### 4.6. Endogenous problem

In order to solve the endogenous problem of this model, we select the performance of the year of M&A, the first year after M&A and the second year after M&A respectively in the benchmark regression part. Regression analysis of the performance of the first year after M&A and the second year after M&A with the data of human capital structure of that year is equivalent to the lag of the explanatory variables for one and two periods, based on the above regression analysis to alleviate the possible endogenous problems in this paper.

## 5. Research conclusions and research recommendations

### 5.1. Research conclusions

We selects the human capital of the enterprises to study its impact on the performance of international M&A, and adds cultural differences as a regulatory variable. After empirical research, the following conclusions were drawn:

(1) In the year of M&A, employee compensation was positively correlated with the company’s international M&A performance, and passed the significance test.
(2) The impact of employee training on the performance of corporate international M&A has not passed the significance test.
(3) In the year of M&A, the relationship between employee education level and the company’s international M&A performance is positively correlated, and the significance test is passed.
(4) In the year of M&A, the impact of employee turnover on the performance of corporate international M&A was negatively correlated and passed a significance test.
(5) In the year of M&A, the impact of employee technical personnel on the performance of corporate international M&A was significantly positively correlated and passed a significance test.
(6) After introducing the regulatory variables of cultural differences, it can be found that cultural differences can reduce the impact of employee compensation on international M&A performance; while cultural differences have no adjustment effect on the relationship between employee training and international M&A performance; cultural differences are also the same. The relationship between employee turnover and international M&A performance has not been adjusted; for the degree of employee education and the proportion of employees and technicians, cultural differences have not played a regulatory role in the year of M&A. In the second year, cultural differences weakened the negative impact of each of these two factors on international M&A performance.

### 5.2. Research recommendations

We studies the relationship between corporate human capital and corporate international M&A performance, and introduces the cultural differences as a moderator. Through the above research, this paper proposes the following suggestions:

(1) International mergers and acquisitions will affect the vital interests of employees, such as salary, training and development opportunities. After international mergers and acquisitions, enterprises should re-establish a reasonable and sound salary system and training system suitable for enterprise development, appropriately relax salary control and appropriately increase cross-cultural training for employees, reduce the impact on enterprise performance caused by salary rigidity and the unfamiliarity of employees’ work and business caused by mergers and acquisitions, so as to improve the production efficiency of employees, provide basic guarantee for enterprise production development and improve the performance of international mergers and acquisitions.
(2) Enterprises should strengthen their own management and optimize the allocation of human resources after mergers and acquisitions. Introduce highly educated talents, pay attention to the training and development of technical personnel, and try our best to retain key talents, reduce the brain drain caused by mergers and acquisitions, and try our best to maintain the stability of employees. Education level reflects the overall quality and ability of employees, while technical personnel are the core embodiment of enterprise innovation ability. Enterprises should make good use of them in the process of integration, give full play to the learning effect and technical synergy effect of both parties in mergers and acquisitions, and improve the competitiveness of enterprises in the international market.
(3) Cultural differences can moderate the relationship between human capital and international M&A performance. Cultural differences can weaken the positive impact of employee compensation on the performance of international mergers and acquisitions, but also weaken the negative impact of other human capital factors on the performance of international mergers and acquisitions. Therefore, to deal with cultural differences, enterprises should first collect the dominant cultural information of the region where the merged enterprises are located before mergers and acquisitions, understand the cultural differences and cultural conflicts between the two sides. In international mergers and acquisitions, enterprises should actively deal with the unfavorable factors brought by cultural differences to the development of mergers and acquisitions enterprises, combine human resources integration with cultural integration, and make full use of the learning effect and complementary effect between the two sides to make profits internalized.

### 5.3. Further research direction

This paper also has shortcomings in research, which can be further compensated in subsequent studies:

(1) In the study of the performance of international M&A of enterprises, we selects the data of the four years before and after the merger, and does not examine the changes in the performance of international M&A over a longer period of time. The impact of international M&A is a long-term process, especially in the early stages of M&A. Due to lack of experience and other reasons, the performance of M&A often fails to meet expected goals. With time, the performance of international M&A began to improve. Therefore, we does not consider the long-term impact of international M&A performance. It is believed that if the long-term research on international M&A performance can be added, the research results will be more convincing.
(2) As an important part of an enterprise, its human capital is not static and its composition is different at different growth stages of the enterprise. The human capital researched is the composition of human capital in the performance of international M&A, which is considered to be fixed in the short term. If we can join the dynamic process of corporate human capital to study its relationship with the performance of international M&A, it is believed that this will be a more interesting process, and will have a greater guiding significance for reality.

## Supporting information

S1 Data Raw data of the variables involved in this paper.
(XLSX)
